# Implied volatility estimation of bitcoin options and the stylized facts of option pricing

**DOI:** 10.1186/s40854-021-00280-y

**Published:** 2021-09-06

**Authors:** Noshaba Zulfiqar, Saqib Gulzar

**Affiliations:** COMSATS University Islamabad - Wah Campus, Wah Cantt, Pakistan

**Keywords:** Bitcoin options, Deribit, Bitcoin smile, Implied volatility estimation, Numerical estimation

## Abstract

The recently developed Bitcoin futures and options contracts in cryptocurrency derivatives exchanges mark the beginning of a new era in Bitcoin price risk hedging. The need for these tools dates back to the market crash of 1987, when investors needed better ways to protect their portfolios through option insurance. These tools provide greater flexibility to trade and hedge volatile swings in Bitcoin prices effectively. The violation of constant volatility and the log-normality assumption of the Black–Scholes option pricing model led to the discovery of the volatility smile, smirk, or skew in options markets. These stylized facts; that is, the volatility smile and implied volatilities implied by the option prices, are well documented in the option literature for almost all financial markets. These are expected to be true for Bitcoin options as well. The data sets for the study are based on short-dated Bitcoin options (14-day maturity) of two time periods traded on Deribit Bitcoin Futures and Options Exchange, a Netherlands-based cryptocurrency derivative exchange. The estimated results are compared with benchmark Black–Scholes implied volatility values for accuracy and efficiency analysis. This study has two aims: (1) to provide insights into the volatility smile in Bitcoin options and (2) to estimate the implied volatility of Bitcoin options through numerical approximation techniques, specifically the Newton Raphson and Bisection methods. The experimental results show that Bitcoin options belong to the commodity class of assets based on the presence of a volatility forward skew in Bitcoin option data. Moreover, the Newton Raphson and Bisection methods are effective in estimating the implied volatility of Bitcoin options. However, the Newton Raphson forecasting technique converges faster than does the Bisection method.

## Introduction

The emergence of Bitcoin futures and options contracts as cryptocurrencies develop received considerable attention recently. Options and futures contracts are valuable, sophisticated trading tools widely used by investors in traditional markets for speculation and hedging purposes. Keeping pace with the growing market capitalization of cryptocurrency ($ 183.9 billion)^1^ and overnight popularity, an increasing number of innovative derivative instruments (Bitcoin options, Bitcoin futures, and Bitcoin perpetual) were designed to protect potential investors from defined Bitcoin price risk. This ultimately provides a wide range of return opportunities (Deribit 2020). Moreover, well-designed strategies for cryptocurrency derivative instruments improve cost efficiency for potential investors by replacing more capital-intensive strategies (Bitcoin-News 2019). Therefore, taking advantage of the host of opportunities from crypto-market volatility, especially Bitcoin market volatility, the trading of Bitcoin options, futures, and perpetual contracts marks the beginning of a new era.

Bitcoin options are in a nascent stage of development and traded on a handful of Bitcoin futures and options exchanges (Deribit,^2^ LedgerX,^3^ IQ options,^4^ Quedex,^5^ Bitmex,^6^ Bakkt,^7^ and OKEX^8^ internationally to trade Bitcoin futures and options contracts. The recent announcement of the Chicago Mercantile Exchange (CME) group to launch Bitcoin options on Bitcoin futures contracts in the first quarter of 2020 could be seen as a way to help institutions and professional traders in a regulated exchange environment manages spot market Bitcoin exposure, as well as hedge Bitcoin futures (CoinDesk 2019; CME-Group 2020). In this context, Bitcoin options contracts are of immense importance and are now widely adopted and acknowledged by option practitioners, cryptocurrency traders, and policymakers as an effective tool to leverage assets or control portfolio risk by strategically hedging some portion of the risk.^9^

Option valuation plays a fundamental role in managing portfolio returns. It provides a basis for a forecast that assists in rigorous decision making in portfolio management (Pagnottoni 2019). This is particularly true when dealing with the most volatile and immature markets, especially the Bitcoin market. The most popular and widely accepted Black–Scholes option pricing model (Black and Scholes 1973) to determine the fair price of an option (Rebonato 2004; Mayhew 1995) is studied extensively. Options studies are not limited to stock and bonds options; an extraordinarily broad and deep body of the options literature also examines currency options, commodity options, and even interest rate options (Mayhew 1995). The volatility smile, implied volatility surface, and volatilities implied by the option prices are the key phenomena or the stylized facts studied for almost all financial markets globally in the context of option pricing (Jackwerth and Rubinstein 1996; Dupire 1994; Rubinstein 1994; Derman and Kani 1994b; Dupire 1992). Hence, the existence and verification of these phenomena in the option pricing literature motivated us to determine whether we observe the same stylized facts in the most actively traded, and highly volatile, cryptocurrency derivatives market, that is, the Bitcoin options market. Therefore, Bitcoin options could be considered as important as stock, bond, commodity, currency, or interest rate options. To the best of our knowledge, the study of the stylized facts of option pricing for the newly developing Bitcoin options has not yet been addressed. Besides being an area of intense interest, the results of this study would be helpful in defining the appropriate asset class (equity, currency, commodity, etc.) for Bitcoin.

The implied volatilities of Bitcoin options carry important information that is crucial for decision-making process in portfolio management. The closed-form approximations, forecasting ability, and information content of implied volatility is a topic of great interest for option practitioners and academicians. Moreover, producing an accurate and reliable implied volatility forecast is central to derivatives market research, which will be true for the Bitcoin derivatives markets as well. There is an observed absence of root-finding forecasting techniques in the financial literature for estimating implied volatility (Chance et al. 2016). This gap motivated us to estimate the implied volatility of Bitcoin options using root-finding iterative techniques, specifically the Newton Raphson method (NRM)and Bisection method (BM). Notably, this is the first use of numerical approximation techniques to estimate implied volatility for the cryptocurrency derivatives market, to the best of our knowledge.

The data sets for the study emphasize short-dated Bitcoin options (14-day maturity), traded on Deribit Bitcoin Futures and Options Exchange, a Netherlands-based cryptocurrency derivative exchange. To address the issues of generalizability, which requires that we account for the prevailing macroeconomic market conditions, we analyze two different periods: Bitcoin options traded from September 28 2019 to October 11 2019 (dataset-I) and from March 7 2020 to March 20 2020 (dataset-II).

To summarize, we lack a complete understanding of the stylized facts of option pricing (volatility smile and implied volatilities implied by options prices) for Bitcoin options. This study contributes to the cryptocurrency literature and option pricing literature in two ways: (1) we verify the existence of widely accepted volatility smile in Bitcoin options and (2) we estimate the implied volatility of Bitcoin options using the Newton Raphson and Bisection numerical approximation techniques. The results strongly suggest that Bitcoin options belong to the commodity class of assets based on the presence of the volatility forward skew in Bitcoin options data.

We employed Black–Scholes implied volatility ($\sigma _{BMIV}$) as a benchmark for the accuracy analysis (Ewing 2010; Poon and Granger 2003; Isengildina et al. 2007; Li 2005). It is calculated from the pricing error equation $c(\sigma )-c_M=0$; by putting each observable variable into the Black–Scholes option pricing formula and iteratively finding the implied volatility value $(\sigma _{BMIV})$ that satisfies the zero difference between the predicted call premium $c(\sigma )$ and the actual call premium $c_M$. The results show that the newton Raphson and Bisection numerical estimation techniques are effective in estimating the implied volatility of Bitcoin options. However, the Newton Raphson forecasting technique converges faster than does the Bisection method for the at-the-money and out-of-money scenarios.

The remainder of the paper is organized as follows. Section 2 provides a review of the literature on Bitcoin and the estimation techniques to calculate implied volatility. Section 3 presents the research methodology along with the pseudo code of the Newton Raphson method and Bisection method. Section 4 defines the data specifications. Section 5 outlines the stylized facts of option pricing for Bitcoin options. Section 6 describes the implied volatility estimation of Bitcoin options and the pseudo code for the benchmark Black–Scholes implied volatility calculations. Section 7 concludes.

## Literature review

The volatile movement of Bitcoin, exponential growth in returns, unique features, and increasing use worldwide, marks the acceptance of the new crypto-world in recent times (Eross et al. 2019). Bitcoin’s trade trajectory can be traced back to a slice of pizza via a Reddit thread to one of the hottest and debatable commodities in the financial market. Since the inception of Bitcoin by Satoshi Nakamoto (2008) through a ground breaking white paper in 2008, Bitcoin represented the emergence of a new asset class and serves as a diversifier for many investment portfolios due to its low correlation with other traditional asset classes (Burniske and White 2017). In addition, the global investment bank Morgan Stanley^10^ claims to have added more weight to the cryptocurrency profile, stating that Bitcoin is the fastest-growing and best-performing asset class in the last ten years, despite its large volatility swings 2017–2018, as it outperformed many of the best-performing traditional markets, including the S&P 500 index, Dow Jones, and NASDAQ. In this context, Bitcoin is no longer considered simply a payment system or financial system but a preferred choice of institutional investors as an emerging asset class (Burniske and White 2017).

In less than a decade, the cryptocurrency literature grew to cover multiple disciplines by discussing the statistical or economic properties of Bitcoin and providing a detailed overview of the technical issues of Bitcoin and other cryptocurrencies. A rather wide set of studies focuses on the interesting discussion of Bitcoin capabilities as a new financial asset class or an exciting investment opportunity, and whether it exhibits the characteristics of a currency more than a commodity. The majority of the users of Bitcoin treat their Bitcoin investment as a speculative asset instead of considering it as a means of payment (Glaser et al. 2014). Therefore, one can view Bitcoin as a useful asset instead of a currency. In contrast, Whelan (2013) claims that Bitcoin is similar to the dollar in the sense that both have no or limited intrinsic value and can be used primarily as a medium of exchange. The only difference between the two is the centralization of the dollar and the complete decentralization of Bitcoin as it was introduced by the private sector.

Since the inception of Bitcoin, an extensive literature developed in the context of hedging capabilities and the safe-haven properties of Bitcoin in relation to other traditional financial assets based on correlation. An early study on the Bitcoin market by Wu and Pandey (2014) depicted its role in portfolio planning. This study uses the daily prices of Bitcoin and other stock indices for the July 2010–December 2013 period. By analyzing the correlation and volatility of the Bitcoin market, the authors conclude that Bitcoin can best serve as an asset class rather than a currency, and investors can add a portion of this asset to a portfolio to enhance the portfolio efficiency. Baur et al. (2015) examined some specific characteristics of Bitcoin and concluded that Bitcoin is a hybrid of a fiat currency and a commodity and unrelated to other financial assets like equities, bonds, and so on. The study finds that Bitcoin has no intrinsic value and works under an independent, self-governing mechanism. The study also highlighted the role of Bitcoin as a speculative investment and more as an emerging asset class than as a medium of exchange. Dyhrberg (2016a) explored the hedging capabilities of Bitcoin using GARCH models to reveal the relationships between Bitcoin, gold, and the dollar. The results suggest that Bitcoin occupies a place between a currency and a commodity. The reason being the decentralized nature of Bitcoin and limited market size. Moreover, Bitcoin can be seen as a useful tool in portfolio management for making more informed decisions based on its hedging capabilities and for reacting symmetrically to good and bad news. Therefore, Bitcoin can be classified between gold and the dollar, on a scale from the pure medium of exchange benefits to pure store of value benefits.

Dyhrberg (2016b) further investigated the hedging capabilities of Bitcoin against stocks in the FTSE Index and US dollar. The findings suggest a clear place for Bitcoin in the market for portfolio analysis and risk management as a hedge against the FTSE Index and US dollar. Moreover, Bitcoin has some specific speed advantages, including high and continuous frequency trading throughout the week. Therefore, it can be added to an already rich list of hedging tools available to analysts and policymakers to hedge market-specific risk. In response to Dyhrberg (2016a), Baur et al. (2017) replicated and extended the sample period to reveal the relationships between Bitcoin, gold, the dollar, and other financial assets. The study examined the statistical properties of Bitcoin with respect to bonds, stocks, commodities, gold and the US dollar and showed that Bitcoin has distinctively different return, volatility, and correlation characteristics than do other financial assets including gold and the US dollar. The study also showed that Bitcoin is more like an asset than a currency and is used explicitly for speculative investment.

Briere et al. (2015) analyzed a diversified portfolio with Bitcoin along with traditional assets and alternative investments. The exceptional low correlation of Bitcoin with other assets and higher average return and volatility provides significant diversification benefits and may improve the risk-return characteristics of well-diversified portfolios. The low correlation of Bitcoin with other assets may place Bitcoin in the class of safe-haven investments. Bouri et al. (2016) also examined the hedging and safe-haven properties of Bitcoin by using a dynamic conditional correlation model for major world stock indices, bonds, oil, gold, general commodity index, and the US dollar index. The overall results demonstrated that Bitcoin acts as an effective diversifier in most cases. However, the hedging and safe-haven properties may vary between time horizons. This study found that Bitcoin serves as a safe-haven against the weekly down movement in Asian stocks only. Bouri et al. (2017b) examined the hedging capability of Bitcoin under global uncertainty using the first principal component of the VIX for developed and developing markets for different investment horizons. The wavelet and quantile-on-quantile regression estimate results revealed a negative relationship between Bitcoin returns and global uncertainty, leading to the conclusion that Bitcoin can help investors hedge global equity market uncertainty for short time. Selmi et al. (2018) explored the roles of Bitcoin as a hedge, safe-haven, or diversifier compared to gold under extreme oil price movements by utilizing a quantile-on-quantile approach. The findings showed that both Bitcoin and gold would serve as a hedge, safe-haven, and diversifier against oil price movements.

An exhaustive series of studies review the hedging capability and safe-haven property of Bitcoin in contrast with gold, the dollar, and other commodities in recent years. Among them, Shahzad et al. (2019b) addressed the most highlighted question of whether Bitcoin is a better safe-haven investment than gold and a general commodity index by considering several stock market indices in the US, China, and other developing and emerging economies. The author proposes a novel definition of a weak and strong safe-haven after utilizing bivariate cross-quantile algorithm approach. The results revealed the time-varying nature of the safe-haven property for Bitcoin, gold, and other commodities, which differ across the stock market indices included in the study. The study further opened the door by incorporating foreign exchange rates in relation to above-stated markets. Another comparative study by Shahzad et al. (2019a) on the safe-haven and hedging capability of gold and Bitcoin provided great insight for several G7 stock indices. Gold proved to be an undisputable hedge and safe-haven for many G7 stock indices, while Bitcoin served the same purpose for Canada. Moreover, the out-of-sample hedging effectiveness of gold surpasses that of Bitcoin and the conditional diversification benefits of gold in G7 markets are much higher and more stable than those of Bitcoin.

Urquhart and Zhang (2019) analyzed the safe-haven and hedging capability of Bitcoin by accounting for the hourly frequencies of world currencies. Using an ADCC model, the study found Bitcoin to be an intraday hedge for CHF, EUR, and GBP. It acts as a diversifier for AUD, CAD, and JPY and a safe-haven for CAD, CHF, and GBP, even during extreme market conditions. Another study uncovering the hedging and safe-haven properties of eight cryptocurrencies by Bouri et al. (2020a) explored the downside movement of the S&P 500 index and its 10 related equity sectors. The findings indicate that many cryptocurrencies belong to a valuable emerging digital financial asset class. To study the time-varying diversification ability of Bitcoin along with Ethereum and Litecoin against equities, Bouri et al. (2019) provided evidence that all three cryptocurrencies act as a hedge against Asia-Pacific and Japanese equities.

A recent study by Naeem et al. (2020) focused on the safe-haven property and the hedging of the downside risk of commodities considering the functional role of cryptocurrencies. The results suggest the use of Ethereum, Litecoin, and Ripple along with Bitcoin as a feasible hedge and safe-haven against price volatilities in commodities, especially the metals and agricultural groups. These cryptocurrencies are the least effective for energy commodities. Bouri et al. (2020b) examined the same characteristics for Bitcoin, gold, and a commodity index employing a wavelet coherency approach for global and country-specific stock market indices. The results showed overall weak dependence among all, with Bitcoin being the least dependent. The diversification benefits studied through wavelet value-at-risk (VaR) revealed the superior position of Bitcoin over both gold and commodities. Conlon et al. (2020) conducted an interesting study in the context of the recent Covid-19 global Pandemic on the safe-haven characteristic of cryptocurrencies for equity markets. The study found that the inclusion of Bitcoin and Ethereum increases portfolio downside risk, proving that these assets were not a safe-haven for the majority of international equity markets during the Covid-19 turmoil, with the exception of the Chinese CSI 300 index. In comparison, Tether acted as safe-haven for all international indices studied.

Bitcoin was also studied extensively in the context of highly speculative and volatile cryptocurrency markets. Baek and Elbeck (2015), Bouoiyour and Selmi (2015), Dwyer (2015), Katsiampa (2017), Bouri et al. (2017a), Pichl and Kaizoji (2017), Ardia et al. (2019) used ARCH-GARCH volatility analysis to explore the time series of Bitcoin. A significant contribution in studying the price dynamics and speculative trading in Bitcoin is made by Blau (2017). Similar to the stock price crash (Wen et al. 2019), speculative bubbles in the Bitcoin market, as illustrated by Cheah and Fry (2015), provides another great insight. Another stream of literature related to Bitcoin price formation includes the work of Madan et al. (2015), Ciaian et al. (2016), McNally (2016). Many studies (Grinberg 2011; Baek and Elbeck 2015; Chu et al. 2015; Lam and Lee 2015; Halaburda 2016; Bariviera et al. 2017; Burniske and White 2017; Trimborn et al. 2019) emphasized the statistical or economic dimensions of Bitcoin. Further studies by Bartos (2015), Urquhart (2011), Nadarajah and Chu (2017) provided important insights on the efficiency or inefficiency of Bitcoin in terms of the efficient market hypothesis. Shaikh (2020) studied the effect of economic policy uncertainty in the US, UK, Japan, China, and Hong Kong on Bitcoin returns.

As Bitcoin options are at a nascent stage of development, so is the emerging literature related to Bitcoin options implied volatility estimation. Therefore, researchers and practitioners are increasingly interested in the potential to explore the sophisticated tools traded in cryptocurrency derivative markets globally. The recent work on Bitcoin options and futures markets by Geman and Price (2019) highlights the main characteristics of cryptocurrency spot and derivatives markets. This study in particular investigates the storability and convenience yield of Bitcoin. The study further discusses the arbitrage approach to the valuation of Bitcoin and compares the futures and options prices on old and new exchanges.

Machine learning methods are emerging techniques in financial risk analysis (Kou et al. 2014), financial market regulation (Kou et al. 2019), and trade-based money laundering (Chao et al. 2019). Pagnottoni (2019) presents Neural Network Models for implied volatility estimation of Bitcoin options. By highlighting a comparative approach of Neural Network Models with parametric models (binomial and trinomial tree models, finite difference methods and Monte Carlo simulations), the study proves Neural Network Models to be the best estimator of Bitcoin options implied volatility. Moreover, Hou et al. (2019) worked on the pricing of cryptocurrency options and presents the case of Bitcoin and CRIX. The study by Shadab (2014) presented work on Bitcoin regulation and block chain derivatives.

Considering the importance and wide scope of the underlying study, the attempt to precisely estimate implied volatility is central to derivatives market research. This holds equally true for the emerging Bitcoin derivative market. The options pricing literature has several modifications and approximations since the inception of the Black–Scholes option pricing model (Jiang and Tian 2005; Chambers and Nawalkha 2001; Britten-Jones and Neuberger 2000; Beckers 1981; Brenner and Subrahmanyam 1988; Chiras and Manaster 1978; Chance 1996; Corrado and Miller 1996; Bharadia et al. 1996a) to estimate implied volatility with improved precision and accuracy. With the advent of options knowledge, many works study non-parametric models to estimate implied volatility, including Artificial Neural Networks (ANN) (Liu et al. 2019), Genetic Algorithm (Chen and Lee 1997), Back-propagation Neural Network (J.Yao and C.L.Tan 2000), and Sidarto (2006) and the RBF Neural Network (Yan and Jianhui 2016). Jiang (2001) and Berry and Zuo (2009) studied the Bisection algorithm to precisely estimate implied volatility. Unfortunately, all estimates of implied volatility are only an estimate and are subject to an error tolerance and exhibit efficiency issues (Chance et al. 2016). Therefore, Chance et al. (2016) mentioned the absence of precise root-finding forecasting techniques in the financial and options pricing literature for estimating implied volatility.

The present study attempts to contribute to the growing literature on Bitcoin options, cryptocurrency derivatives, and options pricing by exploring the stylized facts of options pricing, considering the volatility smile in the emerging Bitcoin options, specifically traded on Deribit Bitcoin Futures and Options Exchange. This study estimates the implied volatility of Bitcoin options using the Newton Raphson method and Bisection method as numerical approximation iterative techniques. To our knowledge, this study is the first to discuss the stylized pattern of implied volatilities and to use numerical approximation iterative techniques to attempt to estimate the implied volatility of Deribit-exchange-traded Bitcoin options.

## Research methodology

The Black–Scholes–Merton option pricing model (Black and Scholes 1973; Merton 1973) is widely used to determine the fair price of an option. However, the strict set of model assumptions and subjectivity with respect to the parameter choices often yields volatility smiles, skew, smirks, and leptokurtic behavior of the return distributions. These key features are not captured by the simplest Black–Scholes–Merton formula. However, the Black–Scholes–Merton option pricing model is considered to be the cornerstone of options pricing studies (Rebonato 2004). We also employ this traditional model to study the key features of options pricing in a cryptocurrency derivative market, specifically the Bitcoin options market. This model finds the value of an option in a frictionless market using a portfolio of options under arbitrage-free-conditions, as follows:

1 $$\begin{aligned} c&= SN(d_1)-K e^{-r\tau }N(d_2) \end{aligned}$$

2 $$\begin{aligned} d_1&= \frac{\ln (S/K)+(r+(\sigma ^2/2))\tau }{\sigma \sqrt{\tau }} \end{aligned}$$

3 $$\begin{aligned} d_2&= d_1- \sigma \sqrt{\tau }, \end{aligned}$$

where *c* is the call option price, *S* is the Bitcoin price, *K* is the Bitcoin option strike price, *r* is the risk-free interest rate, $\tau$ is the remaining time to maturity, $\sigma$ is the volatility of the Bitcoin returns, and *N*(.) is the cumulative normal density function.

This model can also be used to estimate the volatility of the underlying assets by reverting the process and using the observed market prices for traded call options. By setting the model call price *c* to the observed market price $c_M$, the implied volatility is the implicit solution of

4 $$\begin{aligned} \begin{aligned} c_M&= SN(d_1)-K e^{-r\tau }N(d_2) \\ d_1&= \frac{\ln (S/K)+(r+({\hat{\sigma }}^2/2))\tau }{{\hat{\sigma }}\sqrt{\tau }} \\ d_2&= d_1- {\hat{\sigma }}\sqrt{\tau }, \end{aligned} \end{aligned}$$

where ${\hat{\sigma }}$ is the implied volatility that forces the market observed price to be equal to the model call price.

Because there is no analytical expression to extract the implied volatility from the Black–Scholes formula, many significant studies (Chambers and Nawalkha 2001; Bates 1996; Hallerbach 2004; Kelly 2006; Corrado and Miller 1996; Li 2005) aimed to develop closed-form approximations of implied volatility by relaxing certain assumptions of the Black–Scholes–Merton model. However, the results are not useful for large-scale research and trading (Chance 1996). Some recent works focused on the Genetic Algorithm (Chen and Lee 1997) and Sidarto (2006), RBF Neural Network (Yan and Jianhui 2016), or ANN (Liu et al. 2019) to approximate implied volatility with improved precision and accuracy. These methods require a large amount of training and validation data, which makes it effective only for spreadsheet and pedagogical applications. In this backdrop, Chance et al. (2016) proposed numerical root-finding iterative techniques to estimate implied volatility, which few studies in the finance literature apply to solve such problems. Two well-known root-finding techniques are the Newton Raphson method and the Bisection method (Kritzman 1991). Chriss (1997) and Figlewski et al. (1992) referred to the Bisection method to estimate implied volatility. Other root-finding methods include the Secant method (Chabert 1999), Regula Falsi method (Chabert 1999), and the Dekker–Brent method (Brent 1971) to get accurate estimates of implied volatility.

This study attempts to estimate the implied volatility of emerging Bitcoin options traded on the Deribit Bitcoin Futures and Options Exchange by using the Newton Raphson and Bisection numerical root-finding iterative techniques.

### Root-finding iterative techniques

In scientific or engineering problems, numerical iterative root-finding methods are utilized when the unknown appears implicitly in the formula or the roots of the equation cannot be computed exactly or expressed in closed forms. Mathematically, given a function *f*(*x*), root-finding is the problem of finding a number $x=\eta$ such $f(\eta )=0$. The number $x=\eta$ is a root of equation $f(x)=0$ or a zero of the function *f*(*x*). Linear interpolation is the simplest form of interpolation, which assumes a linear relationship to estimate the function (assumed to be defined by the set of data) at intermediate values. As evident from (4), that there is no linear relationship between the call market price ($c_M$) and the estimated implied volatility (${\hat{\sigma }}$)), so the use of linear interpolation is therefore suspect.

Numerical iterative root-finding techniques start with an initial trial value of the root. It then generates a more accurate estimate in each iteration. The error is computed and the process is repeated with the hope that the sequence will converge to the final root of equation $f(x)=0$. The iterative process may theoretically require an infinite number of steps to reach the solution with 100% accuracy. In practice, an upper bound is defined to terminate the solution.

This study applies the Newton Raphson and Bisection root-finding algorithms to numerically approximate the implied volatility of Bitcoin options from the pricing error equation $c(\sigma _n)-c_M=0$. The solution is considered to converge if the absolute error difference between the current and previous estimate is $< 0.01$. The speed of the algorithm is gauged by noting the count (or the *convergence count*) when the algorithm reaches the tolerance threshold. The maximum threshold for the number of iterations in this study is 1000. For the sake of completion, the next two subsections give a brief overview of the Newton Raphson and Bisection estimation techniques. Readers are referred to Burden et al. (1981), Stoer and Bulirsch (2002), Sidarto (2006), Epperson (2007), Press et al. (2007) for more details.

#### Newton Raphson method (NRM)

The Newton Raphson method is a powerful numerical iterative technique to find the root of equation $g(y)=0$. The estimation begins with a good initial guess. In this study, the initial starting point for the Newton Raphson method is selected according to Manaster and Koehler (1982)

5 $$\begin{aligned} \sigma _0=\sqrt{\frac{2}{\tau }\left\Vert \ln \left(\frac{S}{K}\right)+r\tau \right\Vert }. \end{aligned}$$

The new reference point in each iteration is computed by calculating the zero of the tangent at the previous preference point. Mathematically, it can be explained as follows.

The tangent equation for function *g*(*y*) at a trial value $y_j$ is

6 $$\begin{aligned} g(y)\approx g(y_j)+g'(y_j)(y - y_j)+ \cdots . \end{aligned}$$

The x-intercept of the tangent (i.e., g(y)=0) can be taken as the next approximation ($y_{j+1}$) of the root

7 $$\begin{aligned} g(y_{j+1})=0=g(y_{j})+(y_{j+1} - y_j)g'(y_j). \end{aligned}$$

This implies that

8 $$\begin{aligned} y_{j+1}=y_j-\frac{g(y_j)}{g'(y_j)} ~~~~~j=0,1,2,\cdots . \end{aligned}$$

#### Bisection method

The Bisection method belongs to a family of numerical techniques that use the bracketing method; that is an interval defined by two points, to find the roots instead of relying on point estimates. The main steps of the Bisection method are summarized below.

(i) Define an interval $(x_l,x_u)$ that must include the root of *f*(*x*), where $x_l$ is the lower limit and $x_u$ is the upper limit.
(ii) The mid-point $x_m$ of the interval $(x_m=\frac{x_u+x_l}{2})$ is considered the approximate root.
(iii) if $|f(x_m)|\le \varepsilon$, then $x_m$ is the approximate root. Otherwise, set $x_u=x_m$ if $f(x_l)f(x_m)<0$ or set $x_l=x_m$ if $f(x_l)f(x_m)>0$.
(iv) Repeat steps (ii) and (iii) until the root is reached.

#### Proposed scheme pseudo code

Table 1 describes the pseudo code of the Newton Raphson method and Bisection method algorithms for implied volatility estimation.

**Table 1:**
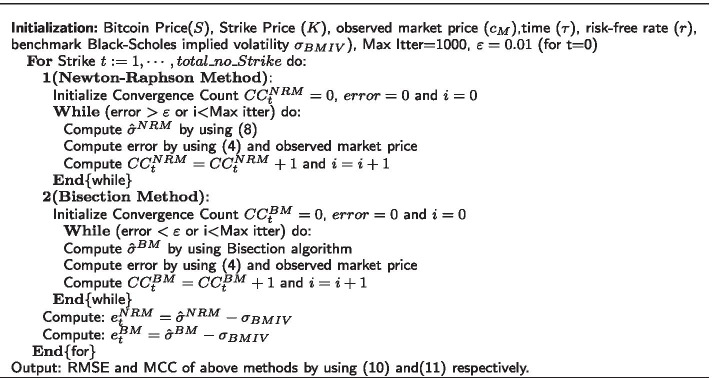
Pseudo code for bitcoin implied volatility (IV) estimation

## Data specification

For the simulation and analysis, we select the Bitcoin implied volatilities (traded on the Deribit Bitcoin Futures and Options Exchange) of 14-day maturity options across a reasonable range of moneyness (strike prices) for two periods: (1) September 28 2019–October 11 2019 (dataset-I) and (2) March 7 2020–March 20 2020 (dataset-II). We selected these periods for the following reasons.

**Fig. 1:**
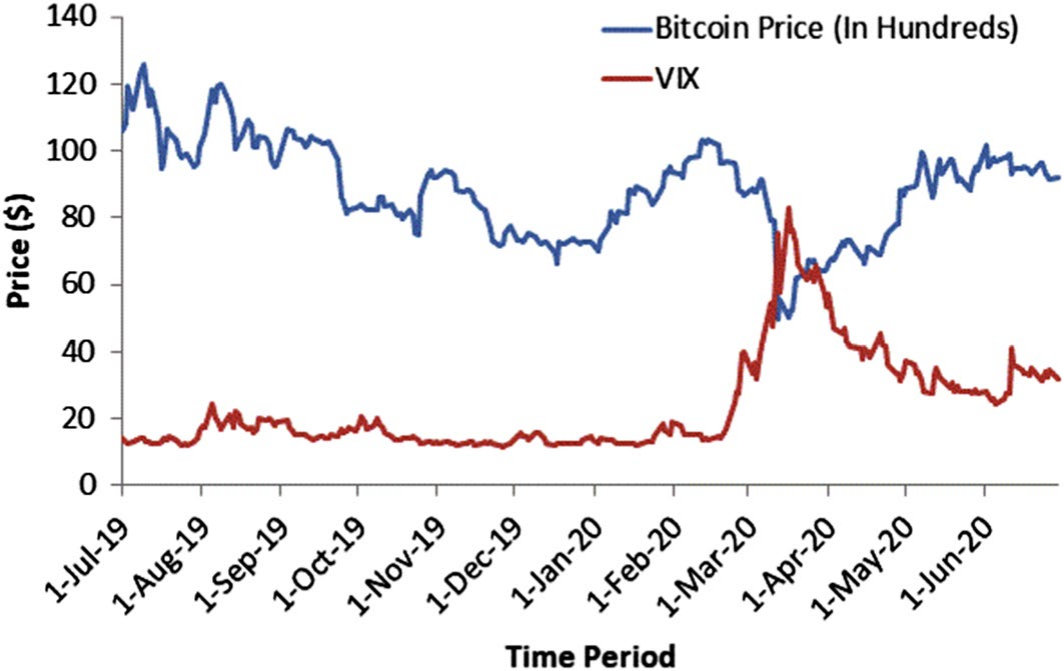
Evolution of Bitcoin price and VIX over time

- To compare the Bitcoin options trading with the movement of the CBOE Volatility Index (VIX). The VIX represents the stock market’s expectation of volatility derived from S&P 500 index options. It is also known as the fear index or fear gauge. Thus, Bitcoin (being the most volatile among all cryptocurrencies) options can be best evaluated in the context of VIX movement. The index was trading at .a low price for dataset-I and at a high price for dataset-II, as shown in Fig. 1. The descriptive statistics for Bitcoin and the VIX over the sample period is given in Table 2.
- To analyze the impact of macroeconomic conditions on the reliability of the estimation techniques applied in this study. The key factors may include Bitcoin price volatility, the volume of Bitcoin options contracts traded, liquidity in the Bitcoin market, Covid-19 global pandemic impact, Bitcoin halving, and so on. Moreover, the expiration of $1 billion worth options contacts on June 26 2020 added much volatility to the Bitcoin market. By considering these market conditions, we can address the issue of the generalizability of the future results to some extent.

**Table 2:** Descriptive statistics of Bitcoin and VIX

Market	Obs	Mean	Median	Std. Dev.	Min	Max
Bitcoin	254	8860	9003	1434.33	4971	12574
VIX	254	23.79	16.54	14.72	11.54	82.69

Bitcoin and VIX price data are obtained from coinmarketcap^11^ and yahoofinance,^12^ respectively, for period of July 1 2019–June 30 2020. It is pertinent to mention that the VIX price data, traded at CBOE, is available for only 5 trading days, whereas the Bitcoin price data includes weekends as Bitcoin market trading is not confined to business days only or to specific trading hours, as in traditional stock exchanges (Dyhrberg 2016b). To show the evolution of these two markets over time, the data are aligned by not considering the Saturdays and Sundays for the Bitcoin market, as evident from Fig. 1.

The Bitcoin option data are collected at 0600 UTC. The interest rates for dataset-I and dataset-II are -0.43% and -0.33%, respectively. The statistical computing program “R” and its contributed package Rmpfr (Maechler 2011) is used for all calculations and simulations of this study.

### Deribit bitcoin futures and options exchange

A handful of cryptocurrency option exchanges to trade Bitcoin options emerged recently, including LedgerX, Bitmex, Deribit, Quedex, Bakkt, and OKEX. Taking advantage of the opportunities of crypto-market volatility, especially Bitcoin market volatility, the Netherlands-based trading platform Deribit Bitcoin Futures and Options Exchange is one of the most liquid and renowned trading platform in the world that offers Bitcoin futures and options contracts.

The Deribit Bitcoin Futures and Options trading platform is an institutional-grade trading platform established in June 2016 to facilitate crypto-traders by offering plain “vanilla” European Bitcoin options and Bitcoin futures with margin. Deribit has ranked in the top 3 crypto-futures exchanges and is the number 1 crypto-options exchange globally, offering European-style options with a right to exercise at the expiration date. It follows the standard Black–Scholes option pricing model to price its actively traded Bitcoin options (Coin-Telegraph 2019b; Hecker-Noon 2018). Approximately 95 % of all trades in 2019 took place on Deribit as an unregulated broker in Amsterdam, the Netherlands. In 2020, it announced that it was moving its operations to Panama, citing regulatory concerns (CoinDesk 2020). Interestingly, Deribit lists standardized Bitcoin options contracts and is the fastest, most technically advanced Bitcoin option exchange to date. In addition, Deribit is the only exchange in the world offering European-style cash-settled options on Ethereum, which it aims to launch very soon. Furthermore, the exchange is planning to introduce innovative instruments like Ethereum and Bitcoin cash futures and perpetual as a first step to enter the Altcoin market.

Classic put-call symmetry (Bates 1999; Rhoads 2011) defines the relationship of call and put options linked by the price of the underlying according to the Black–Scholes–Merton option pricing model. An arbitrage opportunity exists in the market if this relationship does not hold. This opportunity gives sophisticated traders an opportunity to buy or sell stocks immediately to take advantage of mispricing and theoretically generate a risk-free profit.

## Stylized facts of options pricing

The option pricing literature has well documented the presence of two anomalies in the financial data that differ systematically from the Black–Scholes model: (1) a greater degree of excess kurtosis in the unconditional returns distributions and (2) the presence of an implied volatility smile or skew as a result of the excess kurtosis (possibly skewness) in the conditional returns distributions (Das and Sundaram 1999). The options pricing literature also widely acknowledges the term structure of these anomalies, especially the manner in which they change with respect to maturity (implied volatility surface). Hence, some noticeable features that appear to hold across almost all financial markets have been identified (Das and Sundaram 1999; Mayhew 1995).

**Fig. 2:**
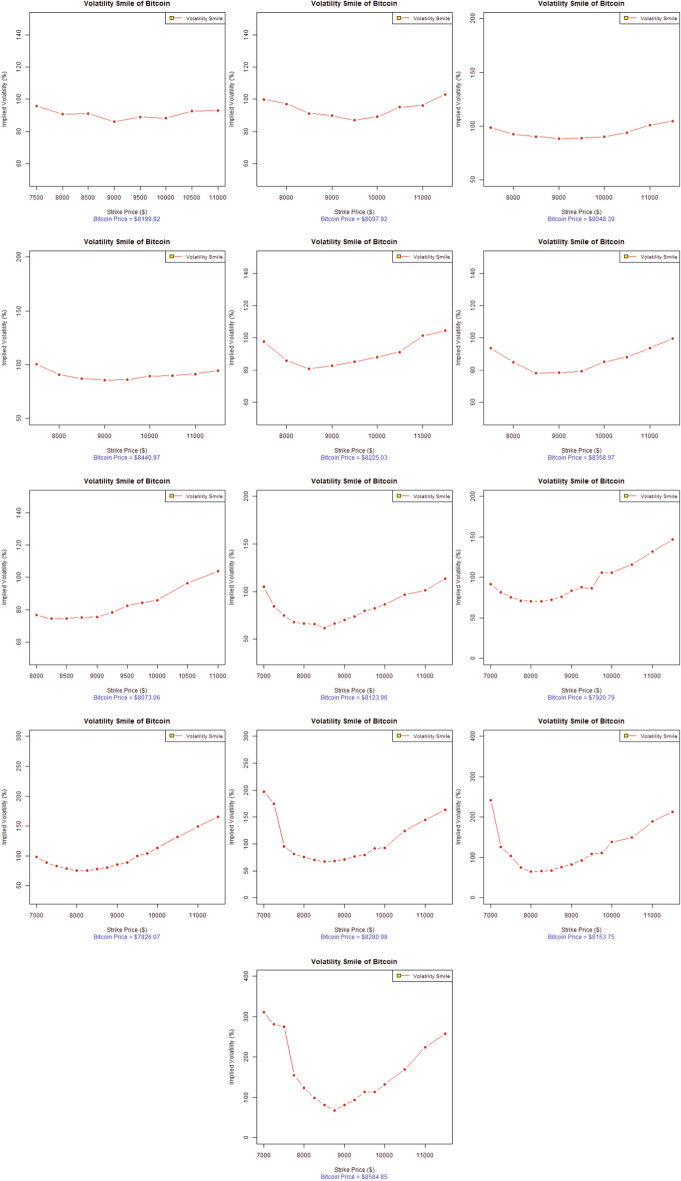
Volatility smile of Bitcoin options (in descending order), 14-Day maturity (dataset-I)

**Fig. 3:**
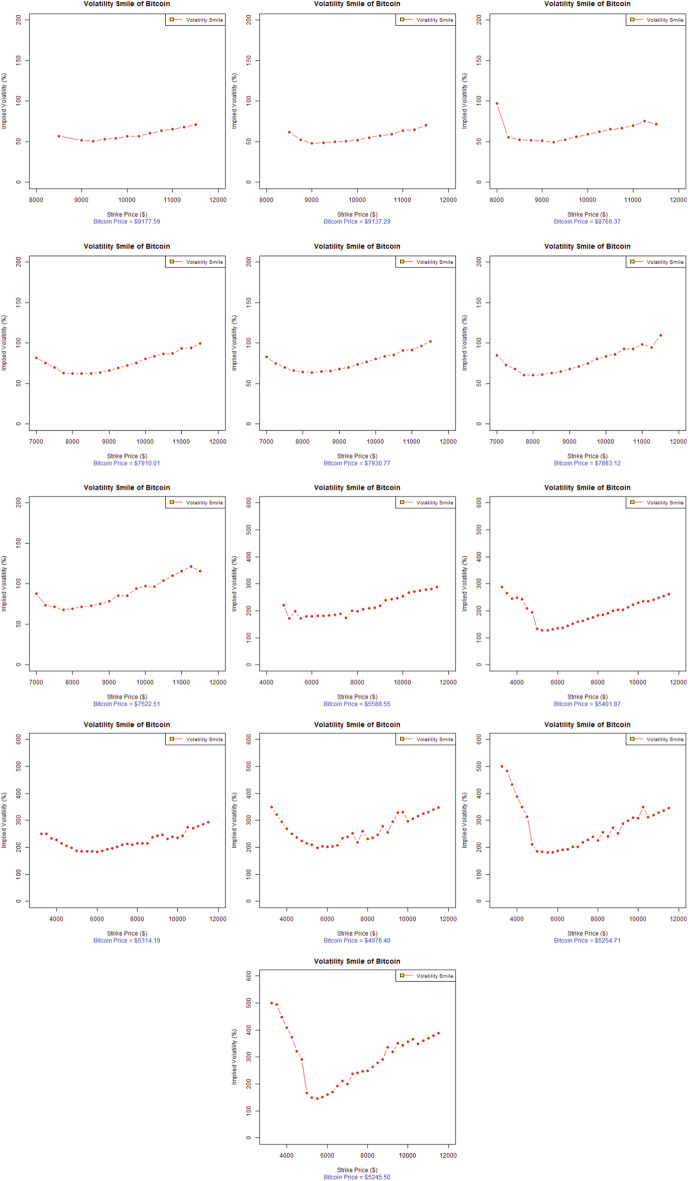
Volatility smile of Bitcoin options (in descending order), 14-Day maturity (dataset-II)

The smile appeared as a post-crash phenomenon in 1987 when implied volatilities were plotted across a reasonable range of moneyness, as reported by Bates (1991), Rubinstein (1994), Ait-Sahalia and Lo (1998), Foresi and Wu (2005), Zhang and Xiang (2008), Corsi (2009). In the context of the Black–Scholes–Merton model (Black and Scholes 1973; Merton 1973), the plot of implied volatility as a function of the strike price (moneyness) should represent a horizontal straight line. This setting implies that all options for buying or selling the same underlying asset with the same expiration date, but with different strike prices, should have the same implied volatilities. However, this was not the case in the actual market data. Thus, the violation of the constant volatility and log-normality of the Black–Scholes–Merton model has given birth to the emergence of the volatility smile, smirk, or skew in the global derivatives markets.

The existing literature identifies a variety of patterns or shapes for the volatility smile including increasing, decreasing, or even non-monotone for almost all financial markets. In-the-money, at-the-money, and out-of-the-money options payoffs play a central role in defining the intrinsic or extrinsic values of options. In-the-money options refer to those with a strike price that is already surpassed by the underlying stock and offers some intrinsic value. In contrast, out-of-the-money options indicate a strike price that the underlying stock has yet to achieve, presenting no intrinsic value at all. At-the-money options refer are those with strike price equal to the underlying stock price, which offer no intrinsic value but may offer some time value (i.e., extrinsic value) (Hull 2006). The implied volatility presents a strong U-shaped pattern when plotted against the strike price; the call option goes from deep-in-the-money to at-the-money and then to deep out-of-the-money, and the put option goes from deep out-of-the-money to at-the-money and then to deep in-the-money as mentioned by Black (1975), MacBeth and Merville (1979), Rubinstein (1985), Derman and Kani (1994a), Shaikh and Padhi (2014).

The existence and persistence of the volatility smile, smirk, or skew in almost all financial markets’ options data is considered to be the strongest empirical regularity. The systematic pattern of the Black–Scholes pricing error across strike prices and maturities was first documented by Black (1975). The study reports that the actual market prices of in-the-money (out-of-the-money) options tend to be lower (higher) than the values given by the Black–Scholes formula. It is now widely acknowledged that the volatility smile is deepest with short-dated (maturity) options in most financial markets and eventually flattens out monotonically for long maturity options (Das and Sundaram 1999).

### Volatility smile of bitcoin options

In the context of the existing literature available on the volatility smile, this study investigates the stylized facts of the volatility smile for the emerging and widely accepted Bitcoin options. Figures 2 and 3 represent the evolution of the 14-day maturity Bitcoin options volatility smile in descending order (from left to right). The results represent two data sets for two separate time periods, in which the VIX was trading at a low price for dataset-I and a high price for dataset-II, as evident from Fig. 1.

Figure 2 indicates that the implied volatilities of Bitcoin options at the lower strike prices are lower than those for the higher strike prices for most of the trading days left to expiry (i.e., 13, 12, 10, 9, 8, 7, 6, and 5 days left to expiry) for dataset-I. The forward volatility skew is quite obvious from Fig. 2. In fact, the forward volatility skew is only the reversed form of the volatility smirk. The forward skew is a particular volatility profile where out-of-the-money calls and in-the-money puts are priced at a much higher implied volatility. This forward skew observed in Bitcoin options suggests that the demand for buying out-of-the-money calls and in-the-money puts dramatically increased for hedging Bitcoin price risk. This is especially true for the trading on the 13th, 12th, 10th, 9th, 8th, 7th, 6th, and 5th days left to expiry, where the Bitcoin options are trading almost around 70% of the implied volatility. Eventually, the implied volatilities rose to more than 130% for most trading days as the Bitcoin options approach expiration and finally account for more than 300% volatility for dataset-I.

Therefore, the most suitable interpretation of the increasing implied volatilities is the demand for these particular strikes of Bitcoin options. Another possible reason for this forward volatility skew in Bitcoin options is a remarkable buying pressure concentrated on out-of-the-money calls, as many institutional investors (Bank of America, Goldman Sachs, JP Morgan, Switzerland’s financial regulation authority, and Nasdaq’s Sweden Exchange) become interested in offering their customized products in exchange for cryptocurrency, preferably transactions in Bitcoin (Investopedia 2020). The increased interest of institutional investors and cryptocurrency practitioners, among many other factors, will ultimately drive the Bitcoin price up. Thus, the implied volatilities of higher strikes will be more elevated than others, as evident from Fig. 2.

Figure 3, illustrating dataset-II, for which the VIX was at an all-time high for 2020 depicts similar characteristics of the volatility smile as Fig. 2. The Bitcoin price data also show the increasing trend for this year, before the Bitcoin market observed a great decline on March 12 2020 from $ 7,650 to $ 5,500, a price drop of approximately 28.10% in market value. This was followed by another abrupt drop to $ 4,679, representing another 14.93% price decline in just one day in the wake of the Covid-19 global pandemic (Coin-Telegraph 2020). Figure 3 illustrates the lower implied volatilities for Bitcoin options at the lower strike prices compared to the implied volatilities at higher strike prices for most of the trading days left to expiration (i.e., 14, 13, and 11–4 days left to expiry). A forward volatility skew is also quite obvious from Fig. 3. Bitcoin options were traded around 50% of the implied volatility at the start and eventually rose to more than 300% for most of the trading days, and finally accounted for more than 500% of the volatility as the Bitcoin options approached expiration for dataset-II.

In the evolution of the implied volatilities for Bitcoin options over 14-days, the volatility forward skew eventually became a symmetrical, more pronounced volatility smile several days before expiration; specifically the 12th or 13th day of options trading, as evident in Figs. 2 and 3. As the implied volatility curve can evolve over time, the volatility smile is the deepest for short-dated options near expiry, as in the options literature (Das and Sundaram 1999). Such movements are of immense importance to both speculators and option and cryptocurrency practitioners, as it suggests that speculators are ready to pour into the volatile Bitcoin market when a volatility smile appears near the expiration. Actually, when the speculative trades approach expiration, there is an increased Bitcoin option trading demand for in-the-money options and out-of-the-money options than at-the-money-options. This higher demand and lack of supply drives the extrinsic value of options upward while increasing their implied volatilities. This is exactly true for the extremely volatile Bitcoin market, where implied volatilities trade from 50% to more than 300%, representing an increase of more than 500% in volatility over 14 trading days. However, the Bitcoin price fluctuated from $ 7,900 to more than $ 8,500, representing an increase of around 7.59% during the 14 trading days in dataset-I. In contrast, the Bitcoin options for dataset-II are more volatile, when the global Covid-19 pandemic added significantly more volatility to the already swinging Bitcoin market. The Bitcoin price moved from $ 8,909.95 to $ 6,198.78, showing a 30.43% decline in the overall Bitcoin price during the 14-day maturity period, touching a new 2020 low as $ 4,679 was a Bitcoin price not seen since April 2019 (Coin-Telegraph 2020).

In analyzing the characteristics of the Bitcoin volatility smile, the presence of the volatility forward skew more closely resembles the skew found in traditional commodity markets than to equity indices or stock options. Based on the analysis and observations, one can conclude that Bitcoin belongs to the commodity class of assets. Referring to the features Bitcoin and commodities share in common, the legitimacy of Bitcoin as a worthy investment, portfolio diversifier, and the best hedging option is induced by the US Commodity Futures Trading Commission (CFTC), which declared Bitcoin to be commodity much like gold, silver, or oil (CNBC 2015). Moreover, the introduction of Bitcoin options in the futures market by the CME in the first quarter of 2020 could be seen as a way forward to deal with the growing demand of practitioners to better manage and hedge Bitcoin exposure in a regulated exchange environment (CoinDesk 2019). Further studies by Baur et al. (2015), Dyhrberg (2016a), Rehman and Apergis (2019) add more weight to our findings in defining Bitcoin asset class among commodities. All-About-Alpha (2019) recently issued a similar observation that Bitcoin derivatives behave much like other underlying assets. We can conclude that Bitcoin options represent similar stylized facts of option pricing as observed in other major financial markets worldwide, as (Bates 1991; Rubinstein 1994; Ait-Sahalia and Lo 1998; Foresi and Wu 2005; Zhang and Xiang 2008; Corsi 2009) report.

### Impact of macroeconomic factors on bitcoin options trading

Several macroeconomic factors added significant volatility to the Bitcoin market and ultimately had a great impact on the trading of Bitcoin options and other derivative instruments during our sample periods. Bitcoin price volatility, the volume of Bitcoin options contracts, Bitcoin market liquidity concerns, Bitcoin halving, the Covid-19 global pandemic impact, the safe-haven and hedging properties of Bitcoin, and the expiration of $1 billion worth of Bitcoin options contacts, contributed considerably to the already fluctuating cryptocurrency market.

After the announcement by the World Health Organization (WHO) declaring Covid-19 a global pandemic on March 12 2020, both the mainstream financial markets and the cryptocurrency market, especially Bitcoin, tumbled drastically in a single day. Although Bitcoin strengthened from $ 4,679 per coin on March 12 2020 to trading around $ 9,132 in June 2020, representing an overall appreciation of 60.57%. The VIX saw a 32.81% decline in value during the same period, as Fig. 1 shows.

**Fig. 4:**
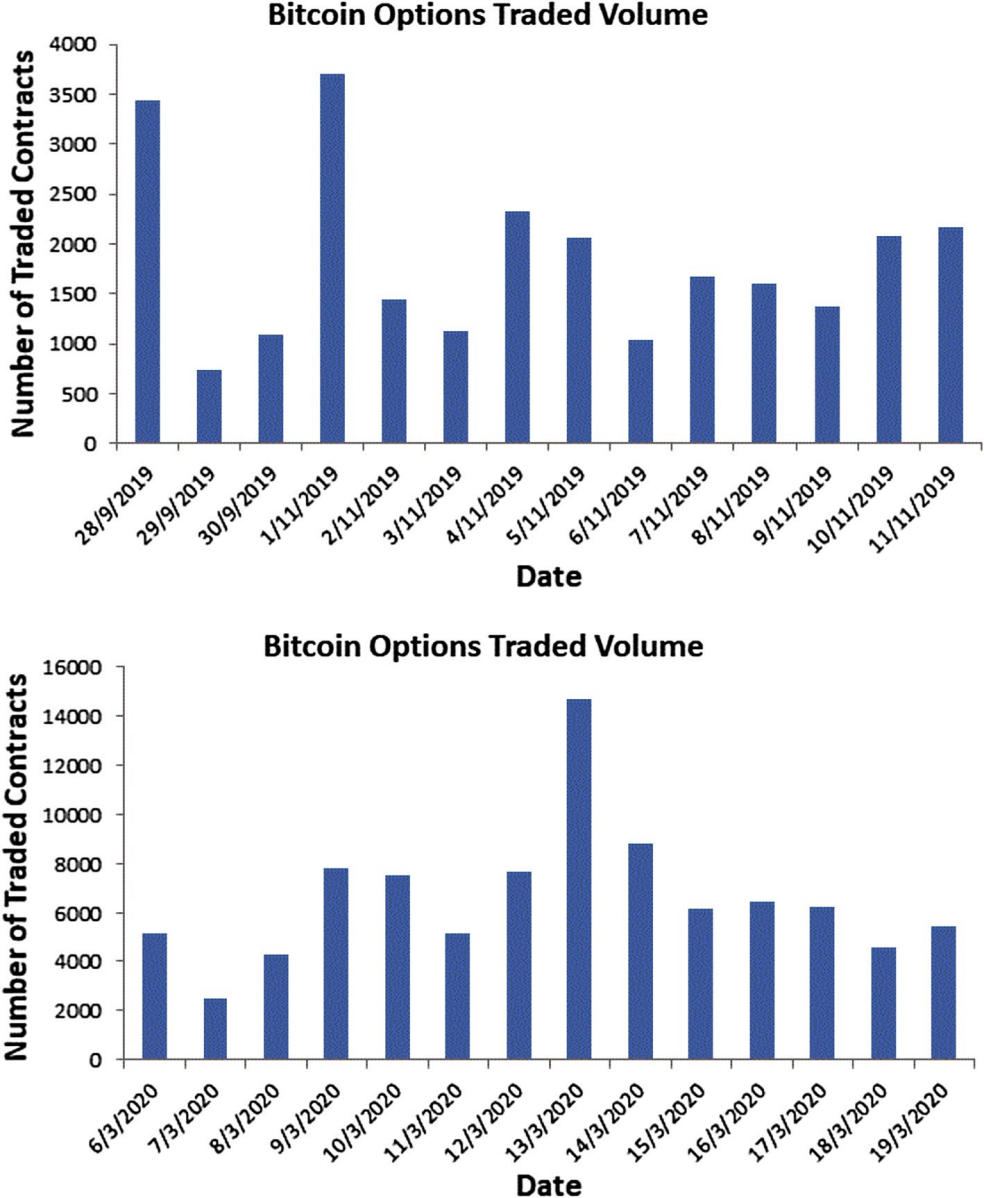
Bitcoin options traded volume for dataset-I and dataset-II

Table 3 and Fig. 4 shows the summary statistics and number of contracts traded (Bitcoin call option volume) for the two datasets in our study.

**Table 3:** Summary statistics of dataset-I and dataset-II

Description	Obs	Mean	Median	SD	Min	Max
Data set-I	14	1845.9	1632.8	873.45	731.5	3703
Data set-II	14	6586	6167	2847.5	2466	14641

The huge spikes of daily volume traded on Deribit are more obvious for dataset-II, as the Bitcoin price declined in mid-March of 2020 in the wake of the Covid-19 global pandemic. Many investors sold off their Bitcoin options to raise cash for margin calls as the number of contracts traded on that particular day reached above 14,000 at one point, as is obvious from Fig. (4).

Bitcoin’s notorious volatility not only scared off large, long-term investors like pension funds, but also attracted hedge funds and high-frequency traders, who take advantage of short-term price moves (Wilson and Carvalho 2020). Moreover, the third Bitcoin halving on May 11, 2020 sparked more interest in Bitcoin options and futures contracts. Bitcoin futures and options contracts traded on Deribit saw massive demand, with open interest reaching $ 1.2 billion in Bitcoin options trading volume compared to the CME ($2.1 million) and Bakkt ($ 1.15 million). Despite the hype created by these new regulated exchanges, Deribit maintained its dominance as an unregulated exchange with 80% of the trading volume in recent times (Bitcoin-News 2020). Furthermore, under the expiry of $ 1 billion worth of options contracts, we would expect that if traders roll over their short positions in June contracts to July and September 2020 contracts, Bitcoin could have observed high price volatility in the following months (Blockchain-News 2020).

However, an interesting debate on the hedging capability and safe-haven property of Bitcoin opened a new avenue in the light of the Covid-19 global pandemic. Investors’ search for shelter against such macro events and many others like shrinking GDP, slowing economic activity, government bailouts, money printing, and unemployment, made the use of Bitcoin and sophisticated Bitcoin options trading an exciting opportunity to mitigate the level of risk by balancing portfolio risk. Being immune to geopolitical tensions, investors are now more prone to add a portion of Bitcoin as an investment to their portfolio as it demonstrated its ability to serve as an economic hedge and safe-haven against unprecedented times (Wu and Pandey 2014; Information-Age 2020). A detailed discussion regarding the safe-haven and hedging properties of Bitcoin is also presented in the literature review section.

Bitcoin technical indicators are tools to predict the direction of Bitcoin price movement. Several indicators exist for Bitcoin trading, such as the Relative Strength Index (RSI), Ichimoku Cloud, Bollinger Bands, Moving Averages, Fibonacci and Volume Indicators, and so on (Bitcoin-Market-Journal 2020). However, the analysis of these indicators on the estimation of implied volatility is beyond the scope of this study.

## Implied volatility estimation of bitcoin options

Options traders use implied volatility as a measure of the market’s opinion of the stock’s potential moves as it provides an informationally superior forecast compared to historical volatility. In addition, it has the capacity to outperform many historical price volatility models (Poon and Granger 2005). In fact, options (calls and puts) are often quoted in terms of implied volatility rather than the price (Poon and Granger 2003; Fengler 2010). Thus, implied volatility estimation and analysis can be crucial in selecting appropriate strategies because the “wrong” implied volatility figures extracted from the “right” market prices could eventually turn into losses of several magnitudes (Rebonato 2004).

The option pricing literature highlights the significance of the option pricing model; that is, relation between the price of an option and the underlying asset price, volatility, and other parameters that influence options process (Mayhew 1995). It further demonstrates the use of historical stock price data to estimate the volatility parameter, which can then be plugged into the option pricing formula to derive options values. Poon and Granger (2003) illustrates that a backward induction technique can be used with the Black–Scholes option pricing model to derive $\sigma$ (volatility of the underlying asset), which the market uses as an input, given that *S* (the price of the underlying asset), *K* (the strike price), *r* (the risk-free interest rate), and $\tau$ (time to option maturity) are observable once the market produces a price (either a quote or transaction price) for the option. This volatility estimate is called the option’s implied volatility.

The extraordinarily broad and deep literature on options pricing elucidates that options pricing formulas often cannot be inverted analytically, so implied volatility must be calculated numerically using various algorithms to find the value of $\sigma$ that makes the price difference equation equal to zero (Mayhew 1995):

9 $$\begin{aligned} c(\sigma )-c_M = 0, \end{aligned}$$

where *c*() is an option pricing formula, $\sigma$ is the volatility parameter, and $c_M$ is the observed market price of the option.

According to Mayhew (1995), the choice of algorithm involves a tradeoff between robustness and speed of convergence. A simple, reliable, but slow approach is to try a series of values for $\sigma$ and choose the one that comes closest to satisfying equation (9). In this context, Ewing (2010) analyzed a Black–Scholes option pricing model that uses equation (9) to find the benchmark implied volatility for an accurate computation of the six implied volatility approximation methods proposed by Curtis and Carriker (1988), Brenner and Subrahmanyam (1988), Bharadia et al. (1996b), Corrado and Miller (1996), Li (2005), and Chargoy-Corona and Ibarra-Valdez (2006). A similar work on comparisons to the benchmark Black–Scholes implied volatility is carried out by Isengildina et al. (2007).

Motivated by this literature, we compute the benchmark implied volatility from the Black–Scholes option pricing model; that is, we plug in all observable parameters and iteratively compute the implied volatility until we find a zero difference between a predicted call premium $c(\sigma )$ and the actual call premium $c_M$. Table 4 describes the pseudo code to find a benchmark Black–Scholes implied volatility for call options, which serves as a basis to compute the mean squared errors of the estimated results through the chosen numerical approximation techniques (the Newton Raphson method and Bisection method).

**Table 4:**
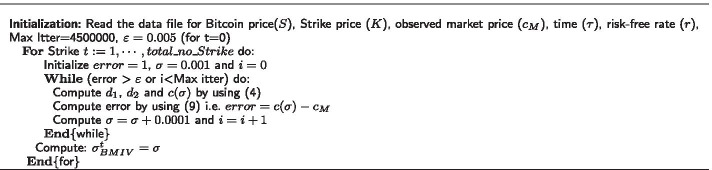
Pseudo code for benchmark Black–Scholes implied volatility ($\sigma _{BMIV}$) calculations for Bitcoin call options

### Simulation setup

To test the effectiveness of the proposed implied volatility estimation techniques, we use the two previously defined 14-day maturity Bitcoin options across a reasonable range of moneyness. The datasets are constructed for two periods based on VIX movement. That is, for dataset-I (-II), the index traded at a low (high) price (see Fig. 1). The Bitcoin options data are collected at 0600 UTC from the Deribit Bitcoin Futures and Option Exchange. The interest rates associated with dataset-I and dataset-II are -0.43% and -0.33%, respectively. Statistical computing program “R” and its contributed package Rmpfr (Maechler 2011) is used for all calculations and simulations.

It is pertinent to highlight that Bitcoin market trading is not confined to business days or specific trading hours, as are traditional stock exchanges, for which studies use daily closing price data for analysis (Dyhrberg 2016b).

### Performance comparisons

**Fig. 5:**
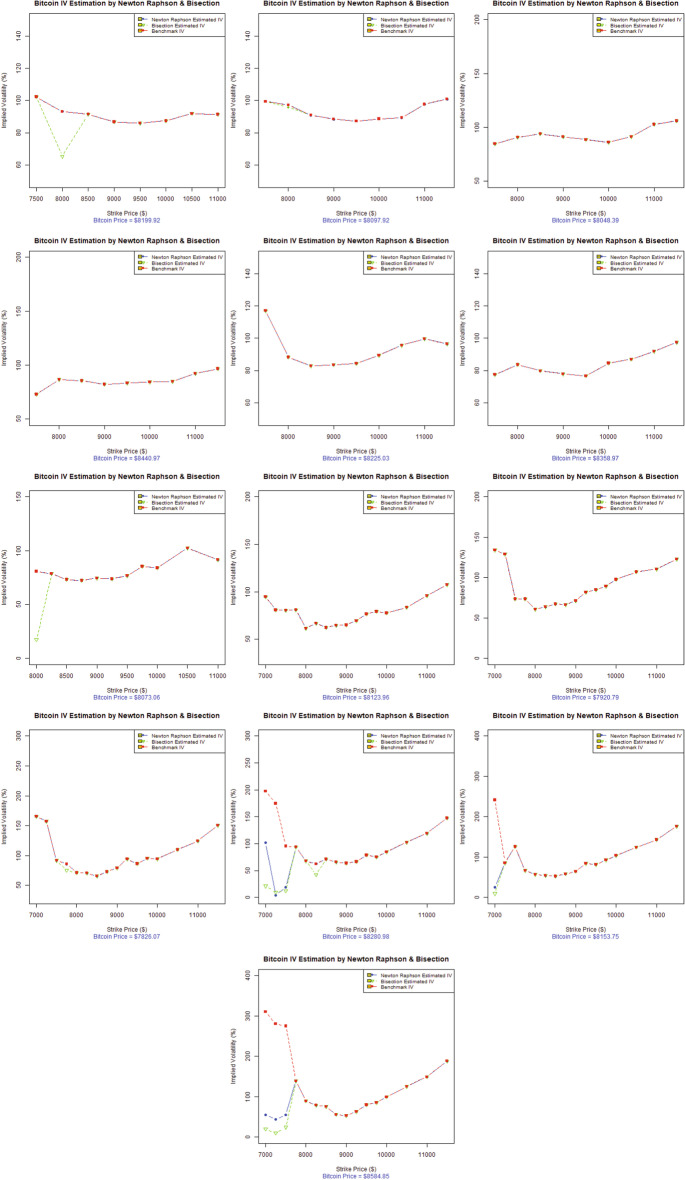
Comparison of Newton Raphson and Bisection implied volatility with benchmark Black–Scholes implied volatility (dataset-I). Graphs are organized in descending order w.r.t date of maturity

**Fig. 6:**
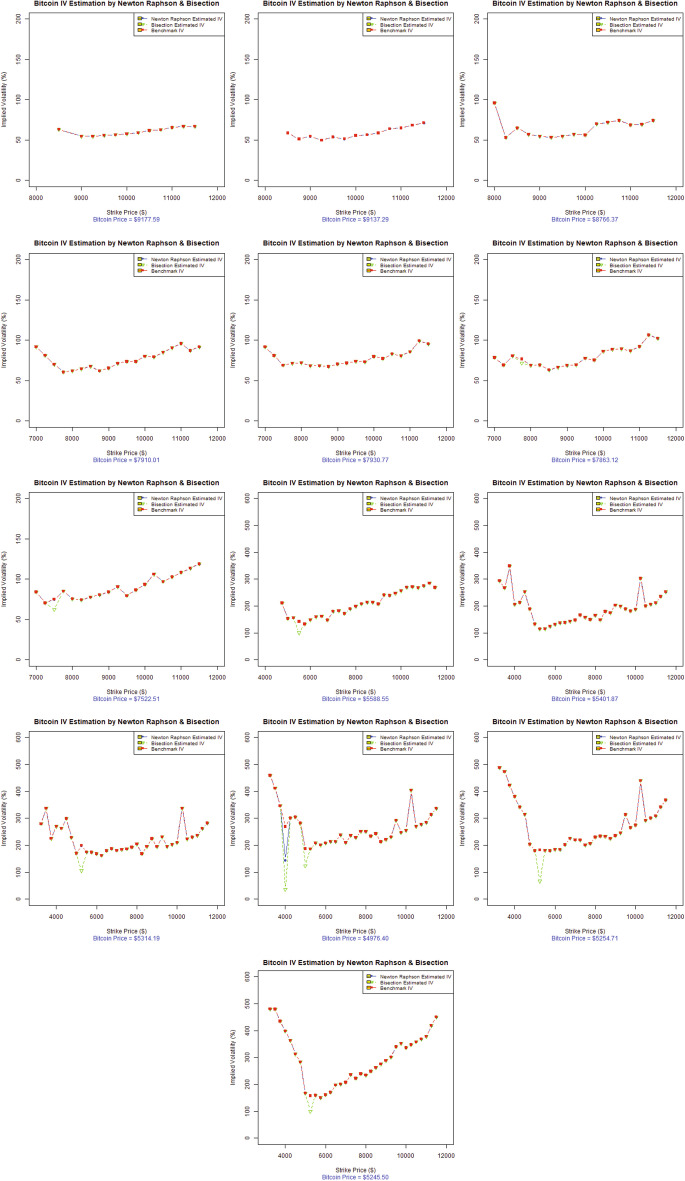
Comparison of Newton Raphson and Bisection implied volatility with benchmark Black–Scholes implied volatility (dataset-II). Graphs are organized in descending order w.r.t date of maturity

To compare the performance of the proposed implied volatility estimation techniques, Figs. 5 and 6 show the Bitcoin options implied volatility versus strike price in descending order of days to maturity for dataset-I and dataset-II, respectively. For qualitative comparisons, the results in Figs. 5 and 6 are marked by a *blue dot-dash line* for the Newton Raphson method, a *green triangle-dash line* for the Bisection method, and a *red square-dash line* for the benchmark Black–Scholes implied volatility. We can explain the varying results of the numerical estimation techniques for the evolution of the Bitcoin volatility smile for in-the-money, at-the-money, and out-of-the-money scenarios as follows.

#### 14–12 Days to maturity

Figure 5(1st row) and 6(1st row) show the estimation for 14-12 days to maturity in descending order. We can see that the Newton Raphson method and Bisection method successfully tracked the benchmark Black–Scholes implied volatilityof Bitcoin options as the call option goes from in-the-money, at-the-money, and deep-out-of-the-money. For dataset-I, some deviation can be observed at the start of the data; however, the estimation improves gradually. For dataset-II, the Newton Raphson method and Bisection method generate a reasonably good estimate throughout.

#### 11–9 Days to maturity

Figures 5(2nd row) and 6(2nd row) illustrate the estimation for 11–9 days to maturity in descending order. Both the Newton Raphson method and Bisection method successfully tracked the benchmark Black–Scholes implied volatility for dataset-1 and dataset-II. We note that for dataset-II, we see a sudden decline of the Bitcoin price in mid-March 2020 in the wake of the Covid-19 global pandemic. However, the Newton Raphson method and Bisection method produce good estimates of implied volatility for both data sets.

#### 8–6 Days to maturity

Figures 5(3rd row) and 6(3rd row) depict the estimation for 8–6 days to maturity in descending order. Some deviations are quite obvious for dataset-II compared to dataset-I. Moreover, for dataset-I, the Newton Raphson method and Bisection method estimations appear robust throughout the call option for the at-the-money and out-of-the-money option scenarios.

#### 5–3 Days to maturity

Figures 5(4th row) and 6(4th row) represent the estimations for 5–3 days to maturity in descending order. The estimated results have small jumps following the benchmark Black–Scholes implied volatility, especially for the in-the-money-scenario for both data sets. However, the estimates improve eventually. It is pertinent to mention that the implied volatilities change into an eminent smile as the days to maturity decrease (especially for dataset-I) and exhibit high implied volatilities for both data sets.

#### 2-Days to maturity

Figures 5(5th row) and 6(5th row) show the estimations for 2 days to maturity. The volatility smile, as mentioned previously, becomes very prominent, providing legitimacy to option pricing and cryptocurrency literature. For dataset-I, the Newton Raphson method and Bisection method have some deviations; however, the estimates improve significantly for at-the-money and deep-out-of-the-money options. For dataset-II, the Newton Raphson method and Bisection method generated a very robust estimate, following the benchmark Black–Scholes implied volatility throughout.

### Performance evaluation

We use two objective measures to quantitatively evaluate the performance of the implied volatility estimation techniques and to compare the results to the benchmark Black–Scholes implied volatility.

**Table 5:** Root mean square error (RMSE) and mean convergence count (MCC) of the estimated and benchmark Black–Scholes implied volatility

DTM	Data set I				Data set II			
	Newton Raphson		Bisection		Newton Raphson		Bisection	
	RMSE	MCC	RMSE	MCC	RMSE	MCC	RMSE	MCC
14	0.001	3.25	0.099	28.88	0.001	3.67	0.001	20.25
13	0.001	3.78	0.003	27.44	0.001	3.77	0.001	20.00
12	0.001	3.89	0.001	19.88	0.001	4.00	0.002	20.07
11	0.001	3.56	0.002	19.44	0.001	4.42	0.001	20.32
10	0.001	3.78	0.001	18.89	0.001	4.63	0.003	19.68
9	0.001	4.00	0.001	18.89	0.001	4.74	0.012	23.89
8	0.001	3.91	0.189	27.27	0.001	5.00	0.029	23.84
7	0.001	4.31	0.001	19.88	0.001	5.21	0.079	22.32
6	0.001	4.38	0.001	19.88	0.001	5.09	0.001	20.12
5	0.001	4.44	0.002	24.00	0.002	5.09	0.159	21.68
4	0.523	4.67	0.639	39.13	0.216	5.15	0.414	26.00
3	0.540	10.31	0.578	24.00	0.001	5.24	0.099	21.79
2	0.446	4.56	1.176	34.25	0.001	5.68	0.099	21.56

#### Root mean square error (RMSE)

The RMSE between the estimated implied volatility and Benchmark Black–Scholes Implied Volatility of Bitcoin options is computed as follows:

10 $$\begin{aligned} RMSE= \sqrt{\frac{1}{N} \sum _{i=1}^N (e_i)^2} \end{aligned}$$

where *N* is the total number of strike prices considered for evaluation, $e_i=(x_i-y_i)$ is the error, $x_i$ is the estimated result, and $y_i$ is the benchmark Black–Scholes implied volatility. Table 5 shows the RMSEs averaged over all strike prices and represent the accuracy analysis of the two algorithms evaluated in this study.

#### Mean convergence count (MCC)

The MCC is calculated as the average of the count the algorithm takes to converge to the implied volatility estimate against each strike price; that is,

11 $$\begin{aligned} MCC=\frac{1}{N}\sum _{i=1}^N |CC_i| \end{aligned}$$

where $CC_i$ is the number of algorithm iterations to settle to final value and *N* is the total number of strike prices evaluated. Table 5 shows the efficiency analysis averaged over all strike prices. The MCC is imperative to the analysis as it tells us how much time an algorithm takes to finally achieve the desired results.

Comparing the results in Table 5, RMSE values are comparable for both the Newton Raphson method and Bisection method in terms of accuracy of the estimation techniques. In contrast, the MCC is much lower for the Newton Raphson method estimation technique than for the Bisection estimation technique. These findings suggest that the Newton Raphson method is efficient for convergence to the desired solution compared to Bisection method for the two data sets studied here. These results are in line with the theory that the Newton Raphson method converges quadrilaterally, while the Bisection method converges linearly (Epperson 2007). Thus, we can infer from Table 5 that the Newton Raphson method has tighter and more accurate estimates and much reduced convergence count than does the Bisection method, especially for the at-the-money and out-of-the-money scenarios.

To summarize, we can conclude from Figs. 5 and 6 and Table 5 that the Newton Raphson method gives encouraging and better estimates of Bitcoin options implied volatility than the Bisection method for most of the trading days for the at-the-money and out-of-the-money options scenarios. However, we observe some deviations or jumps for the in-the-money options scenario. These deviations can be attributed to the choice of algorithm initialization technique. Here, we adopted the technique proposed by Manaster and Koehler (1982). Refining the algorithm initialization scheme for better performance of the in-the-money scenario can be a future avenue to explore.

## Conclusion

The unprecedented rise in the price of Bitcoin over the past decade led to the development of more sophisticated, innovative trading tools like Bitcoin options, futures, or perpetual contracts. Among them, Bitcoin options were designed as a way for hedge funds to manage portfolio risks or to speculate on the price of Bitcoin with quantified risk for better portfolio decision making (Coin-Telegraph 2019a).

This study examined the stylized facts of options pricing for the still-developing Bitcoin options by considering the evolution of the volatility smile for 14-day maturity options at two different periods. Our results demonstrate that Bitcoin options exhibit the same volatility smile characteristics and implied volatility stylized patterns as found in traditional markets (stocks, currencies, exchange rates, commodities, etc.) worldwide (Bates 1991; Rubinstein 1994; Ait-Sahalia and Lo 1998; Foresi and Wu 2005; Zhang and Xiang 2008; Corsi 2009). The empirical results led us to believe that short-dated Bitcoin options tend to produce high volatility as they approach expiration. The practical implication of this phenomenon relates to crypto-options traders who demand more short-dated options, which result in increased buying pressure on the underlying (Bitcoin). This ultimately allows crypto-option traders to charge higher option premiums on Bitcoin call and put options.

Based on our empirical analysis, we can classify Bitcoin options as a commodity-type asset, which provides a significant contribution to the options pricing and cryptocurrency literature in terms of defining Bitcoin’s proper asset class. Moreover, the declaration of Bitcoin as a commodity much like gold, silver, or oil by the CFTC, adds much weight to our findings (CNBC 2015; PYMNTS 2015). Furthermore, the announcement of CME of the launch of Bitcoin options on futures contracts in the first quarter of 2020 provided legitimacy to Bitcoin options trading in a regulated exchange environment. All these factors help push Bitcoin to the center of attraction for policymakers, institutional investors, and even bankers, as these stakeholders cannot ignore its role as a worthy investment or a safe-haven and hedge against major price fluctuations in the market (CoinDesk 2019; Bouri et al. 2020b; Shaikh 2020). This has two important implications for Bitcoin options trading. First, policymakers need to accelerate the global development of cryptocurrency derivatives exchanges that offer a wide variety of sophisticated instruments to hedge against market uncertainties. Second, the regulatory concerns of cryptocurrency derivatives exchanges should be considered at priority.

The study also considers the impact of macroeconomic factors like the Covid-19 global pandemic, the safe-haven and hedging properties of Bitcoin, Bitcoin price volatility, the volume of Bitcoin options contracts traded, Bitcoin market liquidity concerns, Bitcoin halving, and the expiration of $1 billion worth of Bitcoin options contacts, among others, on the estimation techniques we applied in this study. The analytical results demonstrate that the Newton Raphson method and Bisection numerical estimation techniques are effective in estimating the implied volatility of Bitcoin options. However, the Newton Raphson method forecasting technique converges faster than does the Bisection method for the at-the-money and out-of-money options scenarios. Refining the algorithm initialization scheme for better performance in the in-the-money scenarios can be a future avenue to investigate. Numerical estimations of Bitcoin put options is another potential area to study in the future.

## Footnotes

1 (https://www.coinmarketcap.com/)

2 (https://www.deribit.com/)

3 (https://www.ledgerx.com/)

4 (https://www.iqoption.com/)

5 (https://www.quedex.net/)

6 (https://www.bitmex.com)

7 (https://www.bakkt.com)

8 (https://www.okex/en)

9 (https://news.bitcoin.com/)

10 (https://thenextweb.com/hardfork/2018/11/01/morgan-stanley-bitcoin-asset/)

11 (https://www.coinmarketcap.com/)

12 (https://www.yahoofinance.com/)
